# Future Directions in Orthopedic Trauma: From Robotics to Precision Medicine

**DOI:** 10.7759/cureus.114410

**Published:** 2026-08-12

**Authors:** Mohammed Al-Rumaih, Waleed Al-Hoshan, Mansour Al-Shehri, Talal Al-Malki, Salman Al-Harbi, Abdulrahman Al-Otaibi, Meshari Al-Shayie

**Affiliations:** 1 Department of Orthopaedic Surgery, Security Forces Hospital, Riyadh, SAU

**Keywords:** 3d printing, artificial intelligence, orthopedics, orthopedic trauma, precision medicine, robotics

## Abstract

Traditional orthopedic trauma management has been built on standardized fracture classification, surgical fixation principles, and population-based treatment strategies. The limitations of conventional approaches, however, are revealed by persistent issues of malreduction, implant-related complications, fracture nonunion, infection, and variable functional recovery. This narrative review discusses the growing integration of advanced technologies and precision medicine in orthopedic trauma, specifically robotics, artificial intelligence (AI), three-dimensional (3D) printing, and regenerative orthobiologics. A structured literature search was conducted on PubMed/MEDLINE, Scopus, and Google Scholar to find relevant studies published between 2019 and 2026.

The current evidence shows that robotic and computer-assisted systems can increase surgical accuracy, implant positioning, and reduction accuracy in pelvic and spinal surgery, as well as complex fracture fixation. However, there is still no evidence for long-term clinical outcomes. The use of AI in the trauma pathway has shown promising results in fracture detection, automated segmentation, surgical planning, and predicting outcomes. At the same time, challenges remain regarding validation, interpretability, and clinical integration. 3D printing allows for the creation of patient-specific anatomical models, surgical guides, and tailored implants, enhancing preoperative planning and complex reconstruction techniques. Precision medicine strategies that incorporate biomarkers, multi-omics, mechanical phenotyping, and regenerative therapies aim to tailor fracture healing to the patient's biological capacity.

While there has been significant technological advancement, the evidence is still sparse. The future of trauma care will likely be based on a more integrated approach that combines imaging, biomechanics, molecular data, and AI to create treatment pathways that are individualized, adaptive, and outcome-driven.

## Introduction and background

Orthopedic trauma remains a major cause of long-term disability because successful treatment requires considerably more than identifying and stabilizing a fractured bone [1]. In 2021, it is estimated that 172.79 million fractures occurred worldwide, of which 453 million people experienced acute or chronic fracture-related symptoms, and millions of years lived with disability were caused by fractures, despite a slight decrease in age-standardized rates [2]. Although the clinical pathway has improved, with better imaging, implants, and perioperative care, the number of preventable failures remains. These include missed or delayed diagnosis, malreduction, malposition, and cumulative radiation exposure during fluoroscopy-guided procedures [3]. Intra-articular trauma is another challenge, as the initial mechanical trauma can lead to synovial inflammation, chondrocyte death, subchondral-bone remodeling, and progressive cartilage degradation with the development of post-traumatic osteoarthritis despite apparently satisfactory anatomic reduction [4]. These issues are compounded by inequities in access to specialist trauma surgery, advanced imaging, and structured rehabilitation, especially in rural and resource-limited environments [5].

Predicting failure of fracture healing is also challenging. Nonunion is not a single disease entity but a final clinical phenotype produced by interactions between mechanical instability, impaired vascularity, soft-tissue damage, infection, and deficient host biology [6]. A meta-analysis identified infection, open injury, smoking, diabetes, and obesity as independent risk factors for nonunion, as well as greater fracture severity. Population-level risk factors, however, are not yet precise enough for predicting the outcome of a specific patient [7]. Biofilm formation, local vascular compromise, immune dysfunction, and construct stability all play a role in both the eradication of infection and the formation of the bone union, which makes fracture-related infection more complex biologically and mechanically [8]. Chronic pain and restricted mobility can impact independence and employment due to repeated operations. In a large meta-analysis, about 13% of working-age patients became unemployed one year after orthopedic trauma [9].

Traditional surgical treatment of orthopedic trauma includes open reduction and internal fixation with standard plates, screws, and fluoroscopic guidance. Preoperative planning is based on radiographs and CT scans, which forces surgeons to interpret two-dimensional (2D) images and plan the three-dimensional (3D) reduction; digital 3D planning also relies on user experience [10,11]. Intraoperatively, obtaining accurate reduction may necessitate generous exposure and multiple fluoroscopic evaluations, which increases soft-tissue disruption, operative time, and radiation exposure [12]. Moreover, conventional implants are made based on population designs and do not necessarily fit the anatomical variations of every patient, especially in highly comminuted periarticular fractures. Despite improvements in surgical technique, these restrictions may lead to malreduction, implant-related complications, delayed functional recovery, and variable results [10].

These continuing limitations have triggered a simultaneous technological and conceptual transition. Along one axis are enabling technologies such as robotics, computer-assisted navigation, artificial intelligence (AI), and additive manufacturing, all of which seek to minimize human variability, improve geometric accuracy, and decrease radiation exposure [13]. Along the parallel axis are the principles of precision medicine, individual anatomy, biomechanics, molecular and cellular healing capacity, kinematics, and recovery trajectory, which aim to tailor interventions to the biology of the individual patient rather than to a generic fracture classification [10,14]. This review aims to critically review and synthesize emerging technologies and precision-medicine approaches in orthopedic trauma and how these might contribute to safe, equitable, and outcome-driven precision care in orthopedic trauma.

## Review

Methodology

For this review article, the structured literature search involved searching for the latest peer-reviewed journal articles published between 2019 and 2026, using PubMed/MEDLINE, Scopus, and Google Scholar. The search focused on the latest clinical, translational, and technological advances in robotics, AI, digital surgery, fracture-healing prediction, smart implants, regenerative medicine, and precision orthopedic trauma.

A combination of Medical Subject Headings (MeSH) and free-text keywords was used in the search strategy. The MeSH terms used were “Fractures, Bone,” “Fracture Fixation, Internal,” “Artificial Intelligence,” “Robotics,” "Precision Medicine,” “Three-Dimensional Printing,” “Regenerative Medicine,” and “Biomarkers.” These terms were used in the main search string, which was combined with Boolean operators: (fracture OR orthopedic trauma) AND (robotics OR artificial intelligence OR machine learning OR 3D printing OR precision medicine OR regenerative medicine OR biomarkers).

Articles were initially screened through titles and abstracts, and then full-text articles were assessed for possible inclusion in the study. Studies were selected if they were peer-reviewed, published in English, and directly related to orthopedic trauma diagnosis, surgical treatment, fracture healing, complications, rehabilitation, or future precision-care models. Findings were synthesized narratively by technological development, clinical use, the strength of the evidence, and future potential.

Results and discussion

Robotics in Orthopedic Trauma

The introduction of robotic systems in orthopedic trauma came primarily through the adaptation of platforms used in spine and arthroplasty. Optical tracking systems, including active guidance and semi-active guidance, as well as preoperative and intraoperative registration, were among the first to be used with percutaneous screw placement in pelvic ring injuries, femur neck fractures, and thoracolumbar trauma [15]. More recently, however, purpose-built high-force platforms have been developed specifically for long bone and pelvic reduction. Usually, such systems use six-degree-of-freedom (6-DOF) parallel mechanisms that can produce the large muscular forces around the femur or the pelvis, which are necessary to generate traction and torque.

One of the most clinically validated uses of robotic assistance in trauma surgery is the fixation of screws into the pelvic and sacroiliac joints, where the aim of the screw trajectory is a technical challenge with potential for neurovascular injuries [15,16]. Zhao et al. conducted a meta-analysis that showed that the accuracy of screw positioning and amount of fluoroscopic exposure during sacroiliac screw placement were improved with TiRobot-assisted surgery. A systematic review by Wu et al. (2025) on robot-assisted pelvic and acetabular fracture fixation showed consistently high technical accuracy [17]. Yin et al. (2026) reported good clinical results in the pelvic fracture fixation procedure with the use of robotic assistance [18]. Likewise, Al-Naseem et al. found that robotic-assisted percutaneous sacroiliac fixation (P-SIF) demonstrated greater fixation accuracy and fewer guidewire placement attempts compared with traditional methods [19].

Fan et al. (2026) showed that the median residual displacement was 4.0 mm on postoperative CT scan and the rate of reduction was excellent to good (90.6% by Matta criteria) in a series of 32 Tile B/C injuries, with no major neurovascular or implant-related complications and a mean Majeed score of 76.7 [20]. However, experimental and early clinical studies by Gaytan et al. (2026) have shown even tighter control with parallel-robot systems for femoral shaft fractures. For the validation of the Robossis 6-DOF parallel platform, the authors reported sub-millimeter translational errors and sub-degree rotational errors (75th percentile <1 mm and <1°) across clinically relevant trajectories [21]. In comparison, the prospective controlled comparison of robot-assisted and conventional closed reduction of femoral shaft fractures by Zhao et al. (2025) reported significant reduction in operation postoperative length discrepancies (1.74 mm vs. 4.16 mm), postoperative anteversion differences (3.66° vs 13.81°), and a significant reduction of fluoroscopic exposures (Table 1) [22].

**Table 1:** Randomized Controlled Trials (RCTs) Evaluating Robotic-Assisted Orthopedic Surgery THA: total hip arthroplasty; KOOS-12: Knee Injury and Osteoarthritis Outcome Score-12; PCL: posterior cruciate ligament; RAN: robot-assisted navigation; ROSA: Robotic Surgical Assistant

Study	Robot System	Procedure/Indication	Sample Size (Robot/Control)	Main Findings	Complications
Zhang et al. [23]	Robotic-assisted THA platform	Primary total hip arthroplasty	Multicenter parallel-group RCT	• Smaller errors in cup abduction and anteversion • Higher rate of cups in Callanan safe zone • Better limb-length and offset control • Shorter operative time	None
Su et al. [24]	Robotic-assisted navigation	Percutaneous pedicle screw fixation (thoracolumbar fractures)	25/24	• Similar screw accuracy (≈96% Grade A+B) • Markedly lower fluoroscopy frequency (5.2 vs 37.0) • Comparable operative time, blood loss and functional scores	None
MacDessi et al. [25]	Robotic-assisted vs computer-assisted	Total knee arthroplasty (2×2 factorial)	303 total	• No difference in KOOS-12 up to two years • Shorter operating time (-11.5 min) • Better macroscopic PCL soft-tissue score	None
Geng et al. [26]	Novel knee arthroplasty robot	Primary total knee arthroplasty	73/66	• Higher radiographic inlier rate (90.4% vs 59.1%) • Smaller planned-vs-achieved component angle errors	None
Lizcano et al. [27]	Robotic gap-tensioning system	Total knee arthroplasty	30/30	• No difference in Timed Up-and-Go or Stair Climb Test • No difference in KOOS pain or radiographic alignment	None
Zhang et al. [28]	TIANJI Robot system	Displaced midshaft clavicular fractures	Balanced arms (RAN vs plate)	• Shorter operative time, less blood loss, smaller incision • Faster union and higher Constant-Murley score	None
Han et al. [29]	TiRobot	Thoracolumbar pedicle screw placement	115/119	• Higher perfect screw placement (95.3% vs 86.1% Grade A) • Reduced radiation exposure	Low rates of screw-related complications
Möller et al. [30]	ROSA® Knee System	Total knee arthroplasty	77 patients	• Lower frequency of extreme malalignment outliers (>3°) • Improved implant positioning accuracy	None

AI Across the Trauma-Care Pathway

For automated fracture detection on radiographs and CT, the most prevalent method is convolutional neural networks (CNNs) and related deep-learning models [31]. In the review by Dankelman et al. (2023), the accuracy of CNNs used in CT ranged from 69% to 99%, while area under the receiver operating characteristic curve (AUC) ranged from 0.77 to 0.95 [32]. The models in several studies have matched or outperformed unaided clinicians and enhanced overall diagnostic accuracy in a supporting role. Commercial systems like BoneView have demonstrated pooled sensitivities around 92% on multi-regional rib radiographs [33], and dedicated systems for displaced rib fractures had a higher sensitivity than clinicians (97.3% vs 88.2%) [34].

In addition to detection, AI can now aid in automated bone fragmentation segmentation, 3D reconstruction, and virtual reduction. Both the transformer-based and dual-network architectures have been used for complex pelvic and proximal-humerus fractures with mean translational and rotational errors of up to 2.3 mm and 4.5°, respectively [35]. The study by Jeon et al. (2024) verified the clinical feasibility of an AI virtual-reduction model of Neer 3- and 4-part proximal humerus fracture, which achieved reduction plans similar to those planned by experts using the manual method [36]. These tools decrease the time needed for preoperative templating and enhance the accuracy of sizing and trajectory optimization for patient-specific implants [35,36].

New wearable sensors and computer vision models allow for constant tracking of range of motion, compensatory motion, and load-bearing. In simulation, deep-reinforcement-learning (DRL) agents that process inertial and electromyographic (EMG) data can decrease re-injury risk and increase safe return to activity. These closed-loop systems are especially promising for remote, personalized rehabilitation following a trauma [13,35].

Machine-learning models using imaging, laboratory, and clinical information are increasingly used to predict nonunion, length of stay, and other bad outcomes. Early prediction evidence suggests that the AUC for nonunion ranges from 0.83 to 0.86 for ensemble models like XGBoost and Random Forest, but validation is limited [37]. Preoperative-only models have effectively classified prolonged hospital stays from orthopedic procedures based on large national datasets, including factors like procedure type, American Society of Anesthesiologists (ASA) class, and hematocrit [38].

3D Printing and Patient-Specific Surgery

3D printing, also known as additive manufacturing, is an additive fabrication process in which 2D imaging data is used to create a 3D object, layer by layer. Typically, the workflow in orthopedic trauma starts with thin-slice CT acquisition, followed by image segmentation to isolate the bone, fracture fragments, and surrounding structures. The reconstructed anatomy is then converted to a standard tessellation language (STL) file, which can be manipulated in computer-aided design (CAD) software before fabrication [10]. Conventional manufacturing techniques involve material removal via cutting and machining, whereas 3D printing can generate highly customized structures with complex geometries, especially for anatomically challenging fractures, bone defects, and patient-specific implants [10,39,32].

There are several additive manufacturing technologies that are being applied in the orthopedic field. One of the most accessible methods is fused deposition modeling (FDM), which creates models by extruding thermoplastic materials in layers, useful for low-cost anatomical models and surgical education [39]. Stereolithography (SLA) employs a resin that is cured with ultraviolet light to produce highly detailed models with greater surface accuracy, a feature that is useful for complex fracture visualization [39,40]. Selective laser sintering (SLS) and selective laser melting (SLM) involve the use of laser energy to fuse powdered materials. They are beginning to be used for metallic implants, such as titanium patient-specific implants. The electron beam melting (EBM) process is another metal additive manufacturing process that has the potential to produce porous titanium structures having mechanical properties suitable for bone integration (Table 2) [40].

**Table 2:** Different 3D Printing Technologies Used in Orthopedic Trauma [10,39,40] 3D: three-dimensional; FDM: fused deposition modeling; PLA: polylactic acid; ABS: acrylonitrile butadiene styrene; PETG: polyethylene terephthalate glycol; SLA: stereolithography; SLS: selective laser sintering; SLM: selective laser melting; Ti-6Al-4V: titanium-6 aluminum-4 vanadium; EBM: electron beam melting

3D Printing Technology	Key Features and Working Principle	Common Materials Used	Clinical Applications in Orthopedic Trauma
FDM	Extrusion-based technology where thermoplastic filament is melted and deposited layer-by-layer to create a 3D object.	PLA, ABS, PETG, medical-grade polymers	• Low-cost anatomical fracture models • Surgical education and trainee simulation
SLA	Photopolymerization technology where ultraviolet light selectively cures liquid resin to create highly detailed structures.	Photopolymer resins, biocompatible polymers	• High-resolution fracture models. • Detailed visualization of small bone fragments • Preoperative planning for complex periarticular fractures
SLS	Uses a laser to fuse powdered material particles layer-by-layer without requiring support structures.	Nylon, polyamide, polymer composites	• Patient-specific anatomical models • Surgical guides • Experimental orthopedic implants
SLM	Metal additive manufacturing where a high-energy laser completely melts metal powder to create dense metallic components.	Titanium alloys (Ti-6Al-4V), cobalt-chromium alloys	• Patient-specific acetabular implants • Large bone-defect reconstruction • Porous implants for osseointegration
EBM	Uses an electron beam in a vacuum environment to melt metal powder layers and manufacture complex implants.	Titanium alloys, cobalt-chromium alloys	• Custom pelvic and acetabular reconstruction implants • Massive bone-loss reconstruction

Primarily used in trauma surgery, 3D-printed anatomical models have been applied to challenging pelvic, acetabular, calcaneal, and periarticular fractures where 2D imaging may not fully capture fracture morphology. In a meta-analysis of 3D printing-assisted fracture surgery, Yang et al. reported that the surgery was shorter, with less blood loss, and less fluoroscopy use [41]. Likewise, Yammine et al. (2022) reviewed randomized controlled evidence and concluded that 3D-assisted fracture management offered benefits in the perioperative period [42]. A recent study by Long et al. (2025) reviewed the latest 3D printing uses in orthopedic trauma, finding shorter operative times, less blood loss, and better radiological reduction [10].

Precision Medicine in Orthopedic Trauma

A precision approach to fracture healing requires integrating clinical, mechanical, and biological phenotypes. Injury characteristics, such as fracture energy, anatomical location, fracture pattern, gap size, open-fracture classification, contamination, vascular injury, and degree of soft-tissue disruption, should be included in patient-specific assessment. These local factors are coupled with fixation parameters such as construct stiffness, interfragmentary strain, implant configuration, and real-world mechanical loading during rehabilitation [8,14].

Biomarkers are a promising tool for selecting patients who might be at risk for poor healing before they develop radiographic failure. Potential markers of bone formation, bone resorption, inflammation, angiogenesis, and molecular regulation are candidate biomarkers. The markers of bone formation include procollagen type I N-terminal propeptide (P1NP), bone-specific alkaline phosphatase (BAP), and osteocalcin, which indicate osteoblastic activity, while C-terminal telopeptide (CTX) provides information on osteoclastic bone resorption [43]. Some studies have examined whether early biomarker changes predict later union outcomes. A systematic review by Perut et al. (2024) identified the potential association between various circulating bone turnover markers (BTMs) and healing status in long-bone nonunion, including BAP, P1NP, CTX, osteocalcin, and related molecules [44]. Working et al. (2021) found that circulating collagen-X fragments were associated with progression of endochondral fracture repair, suggesting that this biomarker may be useful for identifying early biological changes before clinical radiographic changes [45].

MicroRNAs (miRNAs) play a role in the regulation of osteogenesis, angiogenesis, immune regulation, and osteoclast activity and are now potential regulators and markers of fracture healing. Breulmann et al. (2023) conducted a systematic review of miRNA involvement in fracture healing and nonunion, identifying several promising candidates: miR-21, miR-140, and miR-214 during normal healing and miR-31-5p, miR-221, and miR-451-5p during disturbed healing processes [46]. In the study, Yang et al. (2025) examined new multi-omics applications for bone injury and regeneration. They emphasized the potential to reveal complex molecular networks that regulate fracture repair using these technologies [47]. Precision-trauma platforms of the future could integrate multi-omics data with imaging properties, mechanical parameters, and patient-generated data, alongside AI, to develop dynamic models of fracture-healing trajectories.

Future Directions in Regenerative Medicine and Precision Orthobiologics

Regenerative medicine aims to optimize the healing process of fractured bones by improving the biological environment at the fracture site. Current orthobiological options include autologous bone grafts, bone-marrow aspirate concentrate (BMAC), platelet-rich plasma (PRP), recombinant bone morphogenetic proteins (BMPs), mesenchymal stromal cells (MSCs), bone substitutes, extracellular vesicles, secretomes, and gene-based therapies [48]. While autologous bone graft is the clinical benchmark, the morbidity at the donor site and limited availability have spurred the development of alternative biological therapies [48].

Due to their osteogenic, angiogenic, and immunomodulatory properties, MSCs have been investigated more than any other regenerative therapy. However, the clinical evidence is still much weaker than the preclinical evidence. In the study by Mot et al. (2020), they found inconsistencies in the available clinical studies regarding cell sources, preparation methods, and delivery techniques of MSCs, making it difficult to develop standardized protocols [49]. Yi et al. proposed that the negative impact of poor healing was not observed with MSC treatment, though the changes in healing time were not consistently observed, which leaves questions about the routine clinical use of MSCs [50].

Future strategies focus on precision-based and cell-free technologies, such as extracellular-vesicle therapy, hydrogel-controlled delivery, bioactive scaffolds, immunomodulating biomaterials, oxygen-releasing constructs, vascularized tissue engineering, and regionally administered gene therapy [48,50]. In particular, extracellular vesicles are promising since they carry regenerative signaling molecules without the technical complexity of living-cell implantation. Future clinical models will also incorporate biological profiling, biomarkers, AI prediction, and patient-specific mechanical assessment to determine which patients are more likely to respond to targeted regenerative therapy [50].

## Conclusions

Orthopedic trauma is moving to a paradigm shift from standard fracture care to data-informed precision care. Technologies like robotics, AI, 3D printing, molecular profiling, and regenerative technology offer the potential to advance the accuracy of surgery, predict healing failure, and optimize biological recovery. Current evidence, however, shows greater improvements in technical precision than clinical outcomes over the long term. Future progress will rely on linking mechanical, imaging, biological, and patient-specific information via validated predictive models.
